# Supplementary material for: Cholesterol metabolism promotes B‐cell positioning during immune pathogenesis of chronic obstructive pulmonary disease
Source: EMBO Mol Med. 2018 Apr 19;10(5):e8349. doi: 10.15252/emmm.201708349 (PMC5938615; doi:10.15252/emmm.201708349)
Supplement: Supplementary file 1 — Appendix [file EMMM-10-e8349-s001.pdf]

Appendix

Cholesterol metabolism promotes B cell positioning during immune pathogenesis of chronic obstructive pulmonary disease

Table of contents

Supplementary Methods

Supplementary Methods

Targeted Metabolomics analysis

Targeted metabolomics screening using the Absolute*IDQ*™ p180 Kit (BIOCRATES Life Sciences AG, Innsbruck, Austria) followed by mass spectrometric analysis of lung homogenate was undertaken by the Metabolomics Platform of the Genome Analysis Center of the Helmholtz Zentrum München. The targeted metabolomics approach was based on positive and negative FIA- (flow injection-) and positive LC-ESI-MS/MS (liquid chromatography-electrospray ionisation-triple quadrupol mass spectrometry) measurements by Absolute*IDQ*™ p180 Kit (BIOCRATES Life Sciences AG, Innsbruck, Austria). The assay allows simultaneous quantification of 188 metabolites out of 10 µL plasma, and includes free carnitine (C0), 39 acylcarnitines (Cx:y), 21 amino acids (19 proteinogenic + citrulline + ornithine), 21 biogenic amines, hexoses (sum of hexoses – about 90-95 % glucose), 90 glycerophospholipids (14 lysophosphatidylcholines (lysoPC a Cx:y), 38 diacylphosphatidylcholines (PC aa Cx:y) and 38 acyl-alkyl phosphatidylcholines (PC ae Cx:y)), and 15 sphingolipids including sphingomyelins (SM Cx:y) and hydroxysphingomyelins (SM (OH) Cx:y). The abbreviation Cx:y is used to denote the lipid side chain composition, x and y refers to the total number of carbons and double bonds respectively, as the mass spectrometry technology used cannot distinguish between the side chains of diacylphospholipids. Acyl side chains are abbreviated with an “a”, alkyl and alkenyl residues with an “e”. Side chain substitutions are indicated as follows: hydroxy- (OH), methyl- (M) and dicarboxy- (DC).

The method of Absolute*IDQ*™ p180 Kit has been proven to be in conformance with the EMEA-Guideline "Guideline on bioanalytical method validation (July 21st 2011”), which implies proof of reproducibility within a given error range. Sample preparation and LC-MS/MS measurements were performed as described by the manufacturer in manual UM-P180. Analytical specifications for LOD and evaluated quantification ranges, further LOD for semiquantitative measurements, identities of quantitative and semiquantitative metabolites, specificity, potential interferences, linearity, precision and accuracy, reproducibility and stability were described in Biocrates manual AS-P180. The LODs were set to three times the values of the zero samples (PBS). The LLOQ and ULOQ were determined experimentally by Biocrates.

In detail, tissue homogenate was prepared using a Precellys 24 homogenizer with an integrated cooling unit and homogenization tubes with ceramic beads (1.4 mm): To each mg of frozen lung tissue were added 3 µL of a dry ice cooled mixture of ethanol/phosphate buffer (85/15 v/v). Samples were homogenized at -4 °C for three times over 20 s at 5500 rpm with 30 s pause intervals to ensure

constant temperatures during homogenization. After homogenization, the samples were centrifuged at 4 °C and 2300 × g for 5 min, and the supernatants were used for metabolite quantification. Internal standards for the LC method were applied to the filter inserts of the 96-well kit plate, which already contained the internal standards (ISTD) for the FIA method. 10 µL of each sample (tissue homogenate supernatant, reference plasma, quality controls, zero samples, or calibrators) were added onto the filter inserts and dried for 30 minutes under a nitrogen stream. Amino acids and biogenic amines were derivatized for 20 minutes with an excess of 5% phenylisothiocyanate in ethanol/water/pyridine (ratio 1/1/1, v/v/v), and subsequently dried for 45 minutes under a nitrogen stream. Metabolites and internal standards were then extracted with 300 µL methanol containing 5 mM ammonium acetate by shaking for 30 minutes, and eluted by centrifugation for 5 minutes at room temperature and 500 x g. One half of the eluate was diluted with water (50/50, v/v) for the LC-MS/MS analysis, and the second half of the eluate was diluted with the kits' running solvent (1/5, v/v) for FIA-MS/MS analysis. Both plates were shaken for 5 min at 450 rpm and placed into the cooled auto sampler (10 °C) for LC-MS/MS measurements.

The LC-separation was performed using a mixture of ultrapure water/formic acid v/v 99.8/0.2 as mobile phase A and a mixture of acetonitrile/formic acid v/v 99.8/0.2 as mobile phase B. Amino acids and biogenic amines were separated on the HPLC column Zorbax Eclipse XDB C18 (3 mm x 100 mm, Agilent, product-no. 961967-302) combined with a precolumn SecurityGuard Cartridge C18 (3mm x 4 mm, Phenomenex, product-no. AJO-4287). FIA-analyses were performed using a running solvent that was prepared by mixing 300 mL methanol and the content of an ampule belonging to the p180 Kit. All solvents that have been used for sample preparation and measurement were of HPLC grade.

Metabolite concentrations were determined using internal standards. Except for the sum of hexoses, all metabolites have been analyzed by positive ESI-measurements. For the LC-MS/MS assay, the metabolites were quantified by stable isotope dilution and seven-point calibration curves. Compound identification and quantification were based on scheduled multiple reaction monitoring measurements (sMRM). For the FIA-MS/MS assay, metabolite concentrations were calculated using a onepoint internal standard calibration and are also isotope-corrected. Compound identification and quantification were based on multiple reaction monitoring measurements (MRM). The concentrations of tissue homogenate were reported in µM.

Sample handling was performed by a Hamilton Microlab STAR^{TM} robot (Hamilton Bonaduz AG, Bonaduz, Switzerland) and a Ultravap nitrogen evaporator (Porvair Sciences, Leatherhead, U.K.), beside standard laboratory equipment. Mass spectrometric analyses were done on an API 4000 triple

quadrupole system (Sciex Deutschland GmbH, Darmstadt, Germany) equipped with a 1200 Series HPLC (Agilent Technologies Deutschland GmbH, Böblingen, Germany) and a HTC PAL auto sampler (CTC Analytics, Zwingen, Switzerland) controlled by the software Analyst 1.5.1. Data evaluation for quantification of metabolite concentrations and quality assessment were performed with the software MultiQuant 3.0 (Sciex) and the Met*IDQ*™ software package, which is an integral part of the Absolute*IDQ*™ Kit.
