# Supplementary material for: Cholesterol metabolism promotes B‐cell positioning during immune pathogenesis of chronic obstructive pulmonary disease
Source: EMBO Mol Med. 2018 Apr 19;10(5):e8349. doi: 10.15252/emmm.201708349 (PMC5938615; doi:10.15252/emmm.201708349)
Supplement: Supplementary file 2 — Expanded View Figures PDF [file EMMM-10-e8349-s002.pdf]

Expanded View Figures

Figure EV1. Similar patterns of gene expression in COPD patients and mice exposed to chronic cigarette smoke.

A Heat map of mouse lung and human small airway epithelial cell microarray data (log_2 transformed expression values, $P < 0.05$) following GEO2R analysis of the NCBI GEO data series indicated, with genes grouped according to GO term association. FA, filtered air; CS, cigarette smoke; non-smk, non-smoker.

B Gene set enrichment analysis on the NCBI GEO data series indicated of microarray data from human small airway epithelial cells isolated from COPD patients and smokers (SMK), using gene lists from the Reactome database obtained from the GSEA-Molecular Signatures Database for total TLR and TLR4 specific signaling. NES: normalized enrichment score.

C Representative immunohistochemical analysis of airways from healthy controls, non-emphysematous and emphysematous regions from the lungs of COPD patients, stained to detect TLR4 (brown, arrow) and hematoxylin counter stained. Four patients per group. Scale bar, 100 μm.

D *CH25H* and *TLR4* mRNA abundance in the human bronchial epithelial cell line BEAS-2B treated for 6 h with LPS or CSE at the concentrations indicated ($n = 3$, repeated twice). *CH25H*: *$P = 0.0295$ (1 μg/ml LPS vs. Con), *$P = 0.0347$ (2 μg/ml LPS vs. Con), **$P = 0.0026$ (10% CSE vs. Con). *TLR4*: *$P = 0.0384$ (0.1 μg/ml LPS vs. Con), *$P = 0.0311$ (1 μg/ml LPS vs. Con), *$P = 0.0140$ (2 μg/ml LPS vs. Con).

E *CH25H* mRNA abundance in the human bronchial epithelial cell line 16-HBE treated for 24 h with LPS or CSE at the concentrations indicated ($n = 2$, repeated twice). *$P = 0.0237$ (1 μg/ml LPS vs. Con), **$P = 0.0071$ (10% CSE vs. Con).

F *CH25H* mRNA abundance in the human bronchial epithelial cell line 16-HBE treated for 6 h with TNFα at the concentrations indicated ($n = 3$, repeated twice). **$P = 0.0059$ (50 ng/ml TNFα vs. Con).

Data information: Data are mean ± SD, two-tailed unpaired *t*-test.

© 2018 The Authors

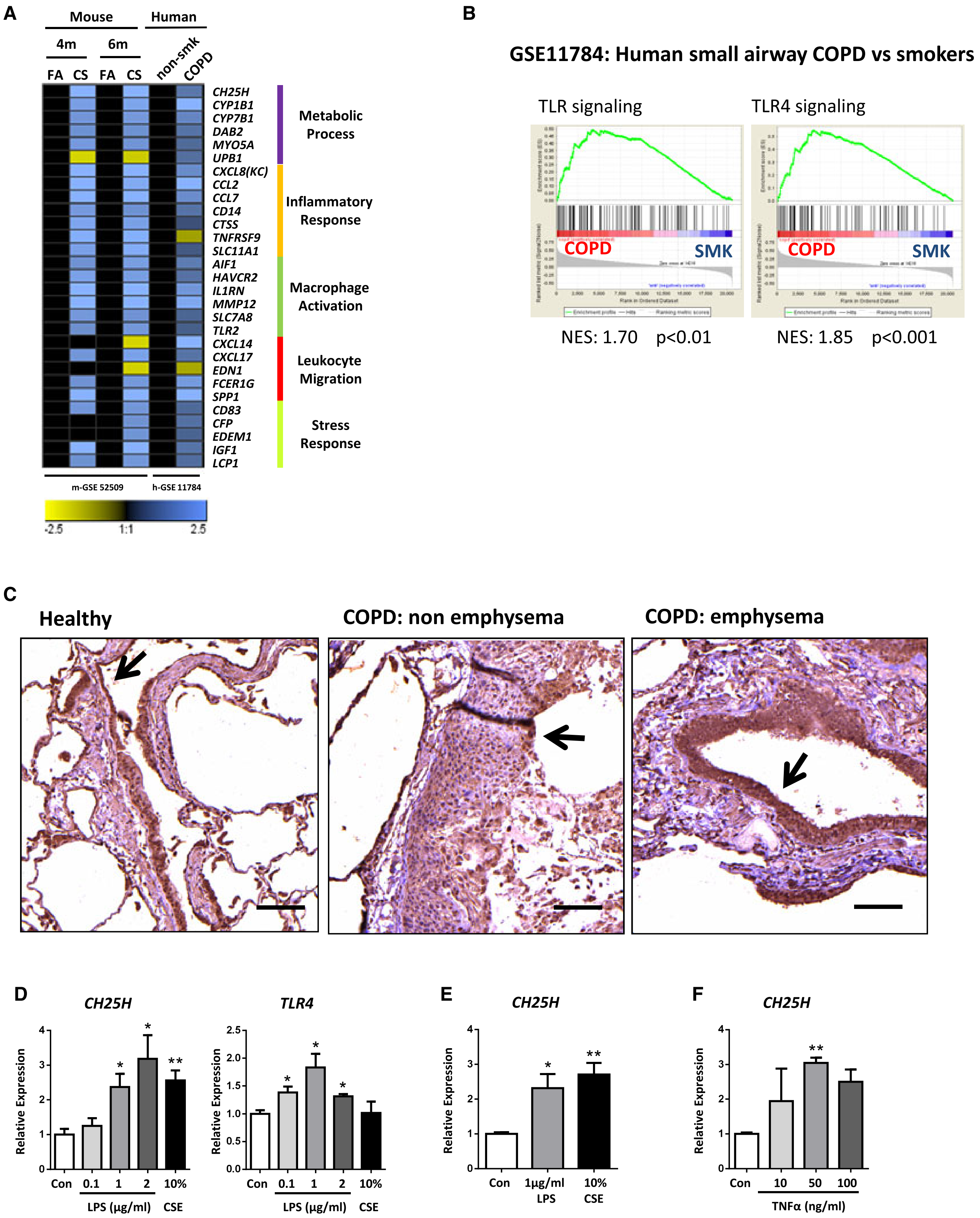
A
Mouse
Human
4m
6m
FA CS FA CS
non-smk
COPD
CH25H
CYP1B1
CYP7B1
DAB2
MYO5A
UPB1
CXCL8(KC)
CCL2
CCL7
CD14
CTSS
TNFRSF9
SLC11A1
AIF1
HAVCR2
IL1RN
MMP12
SLC7A8
TLR2
CXCL14
CXCL17
EDN1
FCER1G
SPP1
CD83
CFP
EDEM1
IGF1
LCP1
Metabolic Process
Inflammatory Response
Macrophage Activation
Leukocyte Migration
Stress Response
m-GSE 52509
h-GSE 11784
-2.5
1:1
2.5
B
GSE11784: Human small airway COPD vs smokers
TLR signaling
TLR4 signaling
COPD
SMK
COPD
SMK
NES: 1.70 p<0.01
NES: 1.85 p<0.001
C
Healthy
COPD: non emphysema
COPD: emphysema
D
CH25H
TLR4
Relative Expression
Con 0.1 1 2 10%
LPS (µg/ml) CSE
E
CH25H
Relative Expression
Con 1µg/ml LPS 10% CSE
F
CH25H
Relative Expression
Con 10 50 100
TNFα (ng/ml)

Figure EV1.

Figure EV2. Metabolic profiles, inflammatory responses, and inflammatory cell recruitment into the lungs of *Ch25h*$^{-/-}$ mice following chronic cigarette smoke exposure.

A Volcano plots of the mean $\log_2$(relative fold change) versus $-\log_{10}$(*P*-value) for each individual metabolite in whole lung tissue as determined by the AbsoluteIDQTM p180 Kit from wild-type (WT) versus *Ch25h*$^{-/-}$ mice. Left: 4-month filtered air (FA) exposure (*n* = 4 mice/group). Right: 4-month cigarette smoke (CS) exposure (*n* = 6 mice/group).

B Heat map of mRNA abundance of the genes shown relative to *Hprt1* and FA controls as determined by RT–qPCR from the lungs of WT and *Ch25h*$^{-/-}$ mice exposed to FA or CS for 4 and 6 months. Data are representative of two independent experiments with four mice per FA group or six mice per CS group.

C Bronchoalveolar lavage fluid total and differential cell counts from the mice described in (B). Upper panels 4 months and lower panels 6 months. Total cells: ****$P < 0.0001$, **$P = 0.0023$ (FA vs. CS, WT mice, 6 m) **$P = 0.0014$ (FA vs. CS, *Ch25h*$^{-/-}$ mice, 6 m). Macrophages: ****$P < 0.0001$, **$P = 0.0015$ (FA vs. CS, WT mice, 4 m), **$P = 0.0077$ (FA vs. CS, WT mice, 6 m), and **$P = 0.0034$ (FA vs. CS, *Ch25h*$^{-/-}$ mice, 6 m). Neutrophils: ****$P < 0.0001$, **$P = 0.0039$ (FA vs. CS, WT mice, 6 m), **$P = 0.0029$ (FA vs. CS, *Ch25h*$^{-/-}$ mice, 6 m). Lymphocytes: **$P = 0.0026$ and ***$P = 0.0005$.

D–F Flow cytometric analysis of whole lung single-cell suspensions gated on $CD45^+$ cells from post-lavaged WT and *Ch25h*$^{-/-}$ mice exposed to FA or CS for 4 months, to detect $F4/80^+$ cells (E) and $CD11c^-$ $CD11b^+$ $F4/80^+$ recruited macrophages (MΦ) (F). $F4/80^+$ cells: **$P = 0.0039$ and ****$P < 0.0001$. Recruited macrophages: *$P = 0.0126$, **$P = 0.0034$ and ****$P < 0.0001$. Data are representative of one experiment with four mice per FA group or six mice per CS group.

G Representative immunohistochemical analysis of wild-type (WT) and CH25H-deficient (*Ch25h*$^{-/-}$) mice exposed to filtered air (FA) or cigarette smoke (CS) for 4 months, stained to detect galectin-3 (red) and hematoxylin counter stained. Four mice per group. Scale bar, 100 μm.

H *Adgre1* (F4/80 gene) mRNA abundance in total lung homogenate from WT and *Ch25h*$^{-/-}$ mice exposed to FA or CS for 4 months. **$P = 0.0045$ and ****$P < 0.0001$. Data are representative of two independent experiments with four mice per FA group or six mice per CS group.

I *Mmp12* and *Timp1* mRNA abundance presented as a ratio in total lung homogenate from WT and *Ch25h*$^{-/-}$ mice exposed to FA or CS for 4 months. **$P = 0.0076$ and ****$P < 0.0001$. Data are representative of two independent experiments with four mice per FA group or six mice per CS group.

J, K Flow cytometric analysis of whole lung single-cell suspensions gated on $CD45^+$ cells from post-lavaged WT and *Ch25h*$^{-/-}$ mice exposed to FA or CS for 4 months, to detect Ly6 g^+ neutrophils. *$P = 0.0106$ (FA vs. CS, WT mice) and *$P = 0.0418$ (CS WT vs. CS *Ch25h*$^{-/-}$ mice). Data are representative of one experiment with four mice per FA group or six mice per CS group.

Data information: Data are mean ± SD. *P*-values determined by one-way ANOVA and Tukey's multiple comparisons test.

 © 2018 The Authors

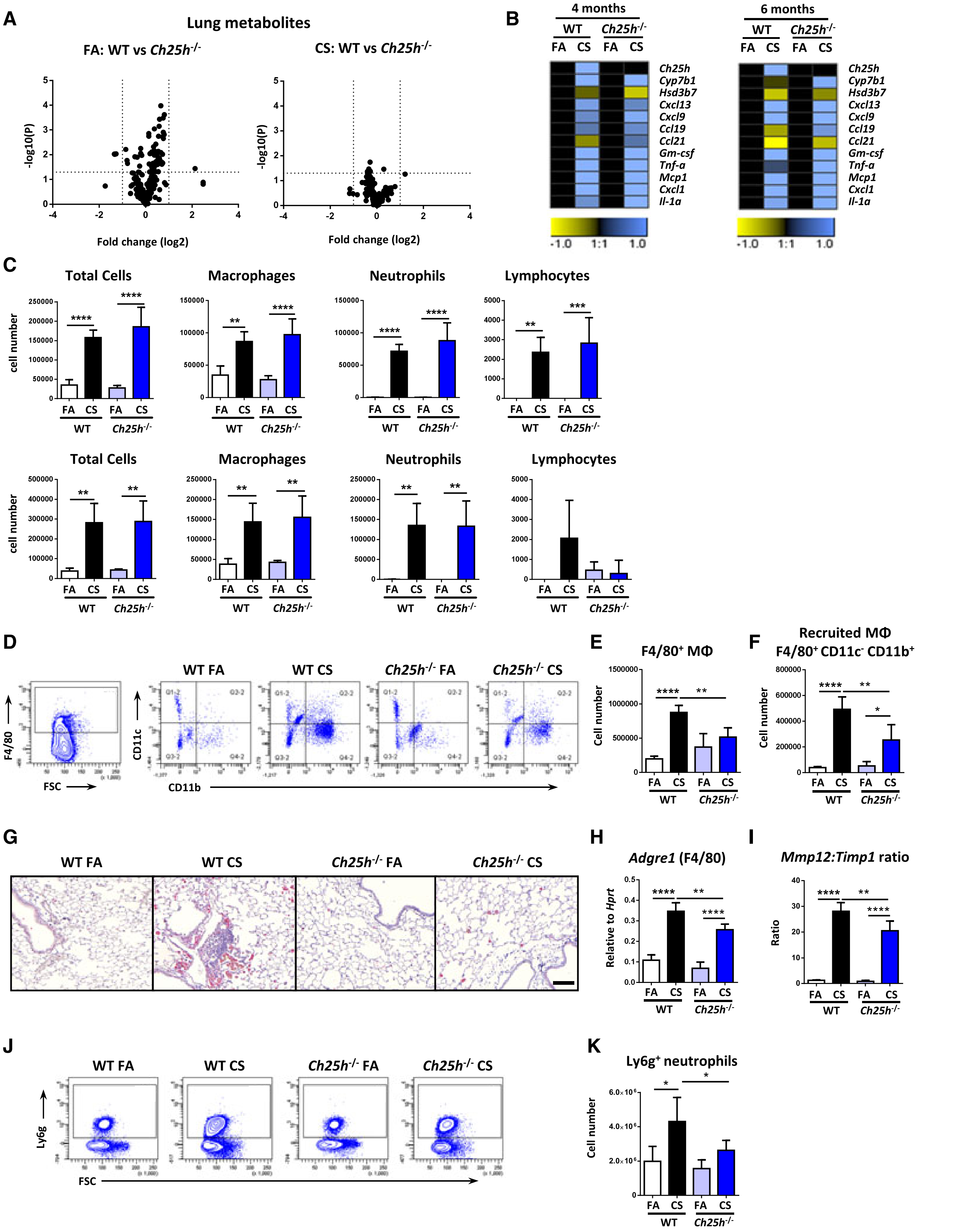
A
Lung metabolites
FA: WT vs Ch25h-/-
CS: WT vs Ch25h-/-
-log10(P)
Fold change (log2)
B
4 months
6 months
WT
Ch25h-/-
FA CS FA CS
Ch25h
Cyp7b1
Hsd3b7
Cxcl13
Cxcl9
Ccl19
Ccl21
Gm-csf
Tnf-a
Mcp1
Cxcl1
Il-1a
-1.0 1:1 1.0
C
Total Cells
Macrophages
Neutrophils
Lymphocytes
cell number
D
F4/80
FSC
WT FA
WT CS
Ch25h-/- FA
Ch25h-/- CS
CD11c
CD11b
E
F4/80+ MΦ
Cell number
F
Recruited MΦ
F4/80+ CD11c- CD11b+
G
WT FA
WT CS
Ch25h-/- FA
Ch25h-/- CS
H
Adgre1 (F4/80)
Relative to Hprt
I
Mmp12:Timp1 ratio
Ratio
J
WT FA
WT CS
Ch25h-/- FA
Ch25h-/- CS
Ly6g
FSC
K
Ly6g+ neutrophils
Cell number

Figure EV2.

Figure EV3. Inflammatory responses and B-cell activation in *Ebi2*$^{-/-}$ mice.

A Bronchoalveolar lavage fluid total and differential cell counts from WT and EBI2-deficient (*Ebi2*$^{-/-}$) mice exposed to filtered air (FA) or cigarette smoke (CS) for 4 months. Data are from one experiment six mice per group (*Ebi2*$^{-/-}$ CS), seven mice per group (WT CS or *Ebi2*$^{-/-}$ FA), or eight mice per group (WT FA). Total cells: *****P* < 0.0001. Macrophages: ***P* = 0.0086 and ****P* = 0.0002. Neutrophils: *****P* < 0.0001. Lymphocytes: ***P* = 0.0087.

B Heat map of mRNA abundance of the genes shown relative to *Hprt1* and FA controls as determined by RT–qPCR from the lungs of WT and *Ebi2*$^{-/-}$ mice exposed to FA or CS for 4 months. Data are from one experiment six mice per group (*Ebi2*$^{-/-}$ CS), seven mice per group (WT CS or *Ebi2*$^{-/-}$ FA), or eight mice per group (WT FA).

C, D Flow cytometric analysis of surface CD69 (C) and MHC class II (D) on splenic B cells isolated from WT and *Ebi2*$^{-/-}$ mice cross-linked with IgM for 18 h. Data from 2 mice with *n* = 2 per mouse. CD69: **P* = 0.0250, ***P* = 0.0020, and ****P* = 0.0006. MHC: **P* = 0.0289 and *****P* < 0.0001.

E *Egr1* mRNA abundance in splenic B cells isolated from WT and *Ebi2*$^{-/-}$ mice cross-linked with IgM for 6 h. Data from 2 mice with *n* = 2 per mouse. **P* = 0.0179, ****P* = 0.0002 (Ctrl vs. IgM, WT), and ****P* = 0.0001 (Ctrl vs. IgM, *Ebi2*$^{-/-}$).

Data information: Data are mean ± SD. *P*-values determined by one-way ANOVA and Tukey's multiple comparisons test.

 © 2018 The Authors

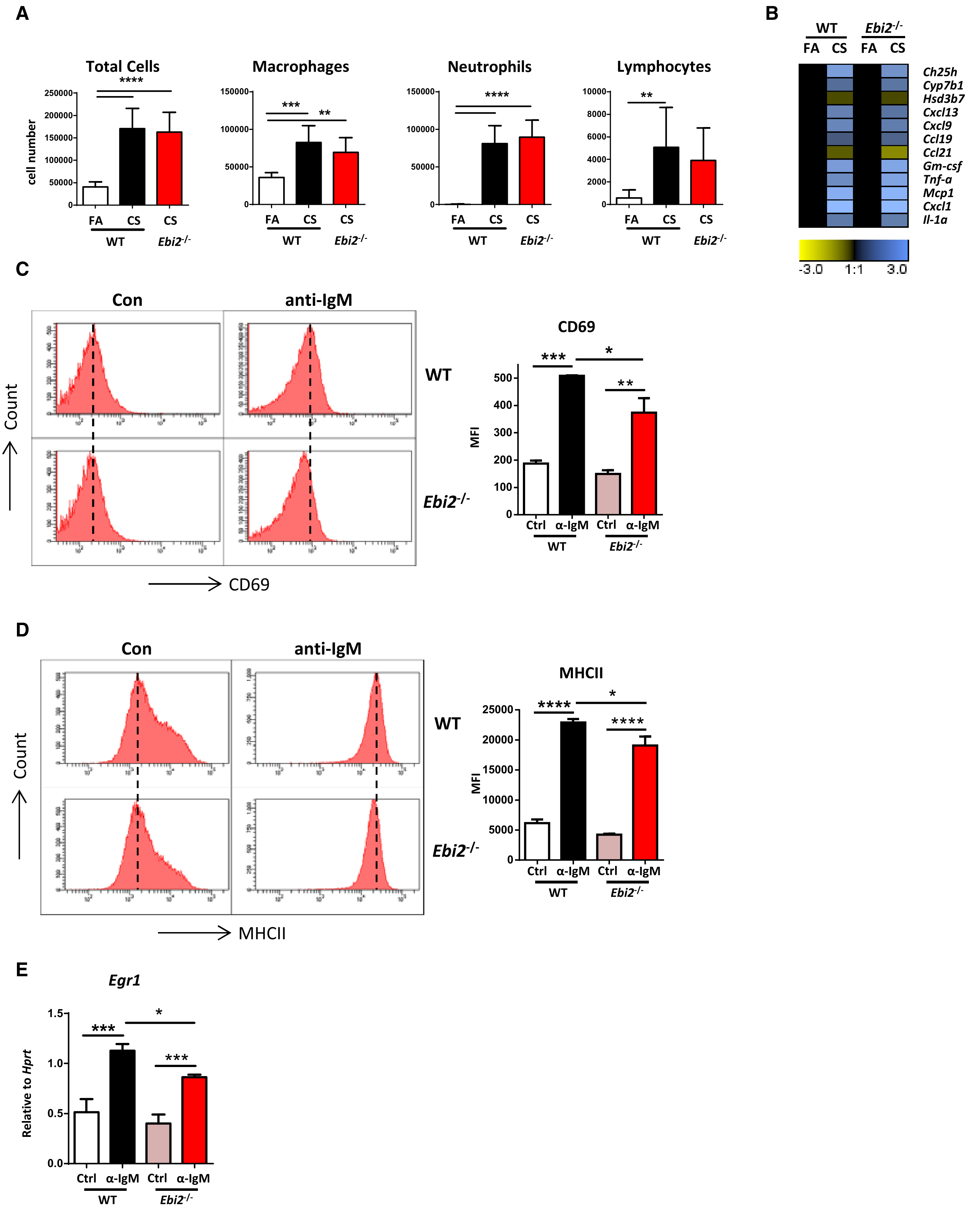
A
Total Cells
Macrophages
Neutrophils
Lymphocytes
cell number
FA CS CS
WT Ebi2-/-
B
WT Ebi2-/-
FA CS FA CS
Ch25h
Cyp7b1
Hsd3b7
Cxcl13
Cxcl9
Ccl19
Ccl21
Gm-csf
Tnf-a
Mcp1
Cxcl1
Il-1a
-3.0 1:1 3.0
C
Con
anti-IgM
WT
Ebi2-/-
Count
CD69
MFI
Ctrl α-IgM Ctrl α-IgM
D
Con
anti-IgM
MHCII
E
Egr1
Relative to Hprt

Figure EV3.

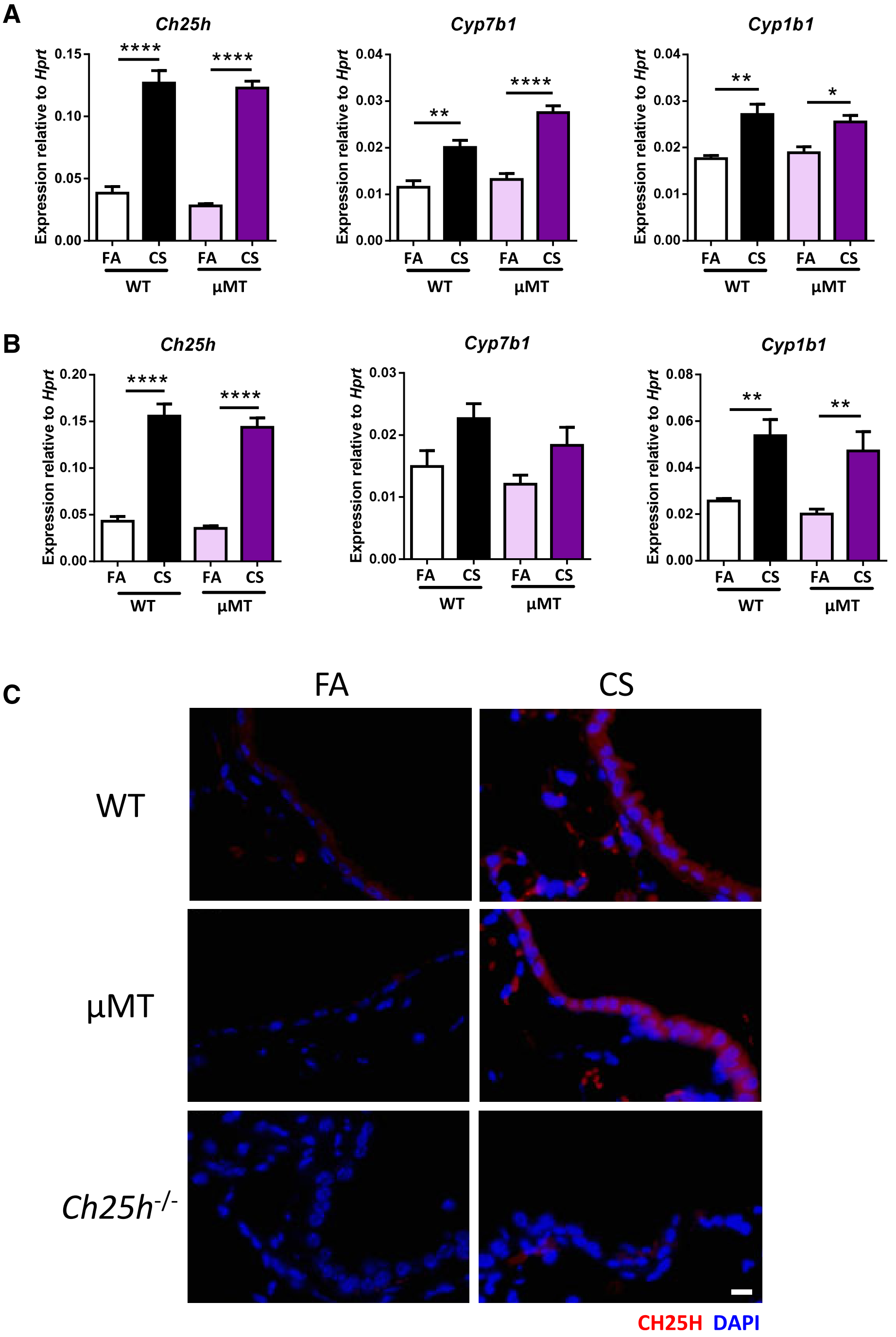

Figure EV4. B cell-deficient mice express similar levels of lipid metabolizing enzymes following acute and chronic cigarette smoke exposure to wild-type animals.

A *Ch25h*, *Cyp7b1*, and *Cyp1b1* mRNA abundance from whole lung of wild-type (WT) and B cell-deficient (μMT) mice exposed to filtered air (FA) or cigarette smoke (CS) for 3 days. Six mice per WT group, and eight mice per μMT group. *Ch25h*: *****P* < 0.0001. *Cyp7b1*: ***P* = 0.0027 and *****P* < 0.0001. *Cyp1b1*: **P* = 0.0117 and ***P* = 0.0052.

B *Ch25h*, *Cyp7b1*, and *Cyp1b1* mRNA abundance from whole lung of WT and μMT mice exposed to FA or CS for 4 months. Experiment repeated twice, eight mice per group. *Ch25h*: *****P* < 0.0001. *Cyp1b1*: ***P* = 0.0063 (FA vs. CS, WT mice) and ***P* = 0.0085 (FA vs. CS, μMT mice).

C Representative immunofluorescence analysis of airway from lungs of WT, μMT, and $Ch25h^{-/-}$ mice exposed to FA or CS for 4 months, stained to detect CH25H (red) and DAPI (blue). Scale bar, 10 μm.

Data information: Data are mean ± SEM. *P*-values determined by one-way ANOVA and Tukey's multiple comparisons test.

 © 2018 The Authors

Figure EV5. Professional APCs isolated from wild-type and *Ch25h*$^{-/-}$ mice are similar and CH25H deficiency does not protect against iBALT-independent emphysema.

A, B *Ch25h*, *Tnfa*, *Il1b*, *Arg1*, *Fizz1*, and *Irf4* mRNA abundance in alveolar macrophages (A) and bone marrow-derived macrophages (B) isolated from wild-type (WT) and *Ch25h*$^{-/-}$ mice polarized under M0, M1 or M2 conditions. *Ch25h*: *P = 0.0129, ***P = 0.0001, and ****P < 0.0001. *Tnfa*: **P = 0.0042 and ****P < 0.0001. *Il1b*: ***P = 0.0002 and ****P < 0.0001. *Arg1*: *P = 0.0107, **P = 0.0025, and ***P = 0.0008. *Fizz1*: *P = 0.0167 and ***P = 0.0003. *Irf4*: *P = 0.0316, **P = 0.0031, and ****P < 0.0001.

C *Ch25h*, *Il12p35*, *Tnfa*, and *Nos2* mRNA abundance in bone marrow-derived DCs isolated from WT or *Ch25h*$^{-/-}$ mice treated with LPS. **P = 0.0048 and ****P < 0.0001.

D Representative H&E-stained lung from wild-type (WT) and *Ch25h*$^{-/-}$ mice treated with a single oropharyngeal application of PBS or porcine pancreatic elastase (PPE) 80 U/kg and analyzed on day 28. Scale bar, 100 μm.

E Mean chord length (MCL) quantification of lung sections from the mice described in (D). **P = 0.0016 and ***P = 0.0002.

F Chord compliance pulmonary function data from the mice in (D). *P = 0.0113 and **P = 0.0052.

G Diffusing capacity for carbon monoxide values from the mice in (D). *P = 0.0295 and **P = 0.0081.

H Bronchoalveolar lavage fluid total and differential cell counts from mice described in (D).

Data information: Data are mean ± SD. P-values determined by one-way ANOVA and Tukey's multiple comparisons test. Data are representative of three independent experiments with seven (A) or five (B) mice per group, or one experiment with four per group (C). In (D–H), data are representative of two independent experiments with four mice per PBS group or five mice per PPE group.

© 2018 The Authors

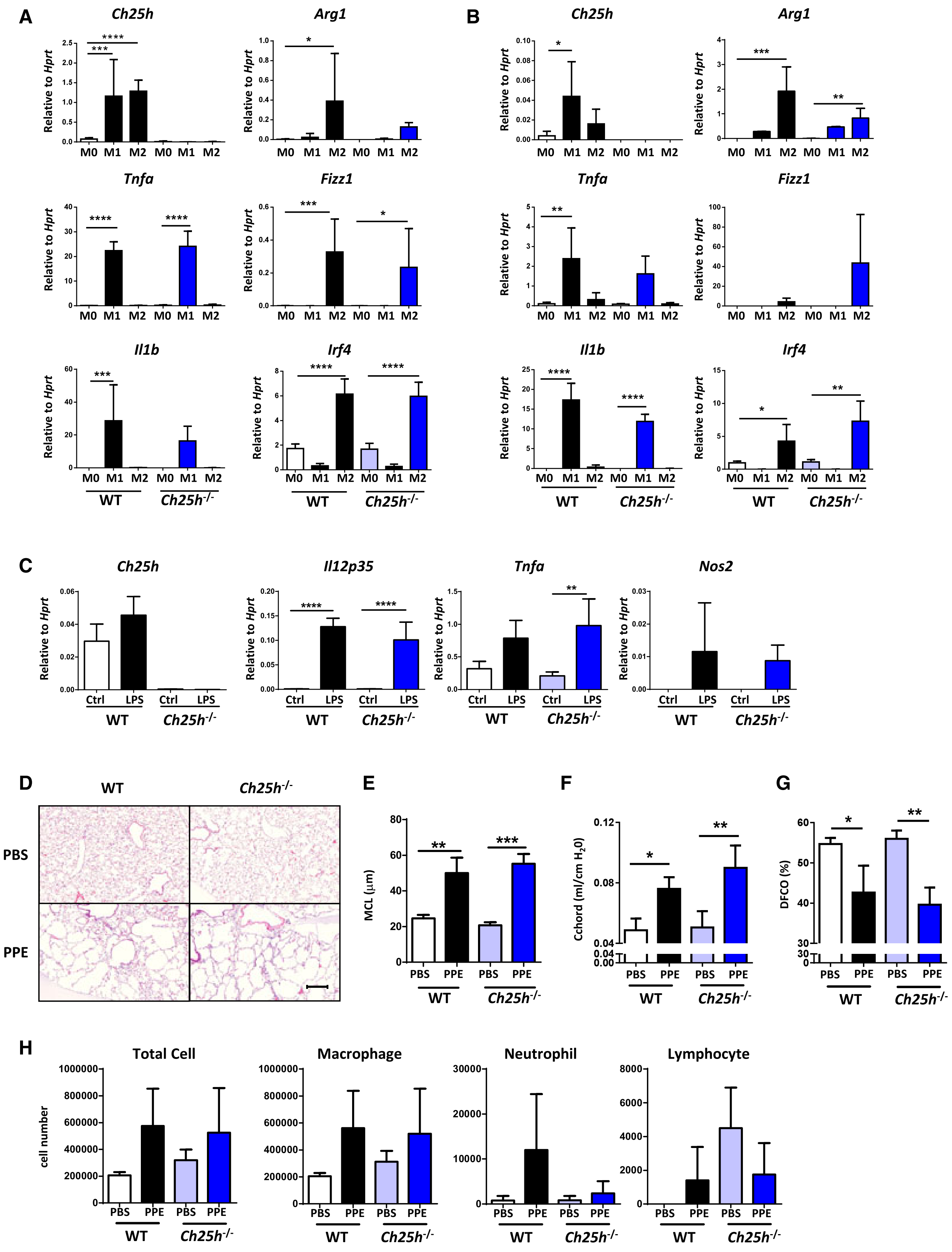

Figure EV5.

 © 2018 The Authors
